# Improving consensus structure by eliminating averaging artifacts

**DOI:** 10.1186/1472-6807-9-12

**Published:** 2009-03-06

**Authors:** Dukka B KC

**Affiliations:** 1 Department of Bioinformatics and Genomics, University of North Carolina at Charlotte, Charlotte, NC, USA

## Abstract

**Background:**

Common structural biology methods (i.e., NMR and molecular dynamics) often produce ensembles of molecular structures. Consequently, averaging of 3D coordinates of molecular structures (proteins and RNA) is a frequent approach to obtain a consensus structure that is representative of the ensemble. However, when the structures are averaged, artifacts can result in unrealistic local geometries, including unphysical bond lengths and angles.

**Results:**

Herein, we describe a method to derive representative structures while limiting the number of artifacts. Our approach is based on a Monte Carlo simulation technique that drives a starting structure (an extended or a 'close-by' structure) towards the 'averaged structure' using a harmonic pseudo energy function. To assess the performance of the algorithm, we applied our approach to Cα models of 1364 proteins generated by the TASSER structure prediction algorithm. The average RMSD of the refined model from the native structure for the set becomes worse by a mere 0.08 Å compared to the average RMSD of the averaged structures from the native structure (3.28 Å for refined structures and 3.36 A for the averaged structures). However, the percentage of atoms involved in clashes is greatly reduced (from 63% to 1%); in fact, the majority of the refined proteins had zero clashes. Moreover, a small number (38) of refined structures resulted in lower RMSD to the native protein versus the averaged structure. Finally, compared to PULCHRA [1], our approach produces representative structure of similar RMSD quality, but with much fewer clashes.

**Conclusion:**

The benchmarking results demonstrate that our approach for removing averaging artifacts can be very beneficial for the structural biology community. Furthermore, the same approach can be applied to almost any problem where averaging of 3D coordinates is performed. Namely, structure averaging is also commonly performed in RNA secondary prediction [2], which could also benefit from our approach.

## Background

Methods for the experimental or theoretical determination of protein structures often output their results as an ensemble. In the case of experimental data like X-ray crystallography data, the ensemble represents both the conformational diversity and the inability to resolve the time and spatial aspects of the experiment, whereas in the case of computational experiments, the ensemble in part represents the uncertainty of interpreting the data[3]. However, often, there is a requirement for a single consensus structure. One way to generate this 'consensus' or 'representative structure' is to calculate the centroid structure by averaging the Cartesian coordinates of the ensemble of superimposed structures.

A series of computational and experimental studies have been performed to rationalize the averaging methodology. Zagrovic et al. [4] proposed the "mean-structure hypothesis" which states that the geometry of the collapsed unfolded state of small peptides and proteins in an average sense corresponds to the geometry of the native structure at equilibrium. Huang et al[5] have shown that finding the "averaged structure" from a set of decoys yield structures that are closer to the native structure than most individual structures. Moreover, Zagrovic et al[6] have shown mathematically that the RMSD between the "averaged structure" and the native structure is more similar than the most individual structures to the native structure. Furthermore, it was also argued that finding average distance matrices and using distance based root mean square deviation as a metric may be one way to capture the relevant features of ensembles of structure and compare them with other reference structures.

Unlike point based averaging where each member is a point, in averaging of structures, the "averaged model" often has unrealistic local geometry, including unphysical bond lengths and angles. In this regard, several methods have been developed to remove averaging artifacts. Due to the process of protein structure prediction, methods to remove averaging artifacts are most commonly developed in this context. The 'regularize' function of REFMAC [7] can be used to regularize the bonds and angles. Furthermore, Betancourt and Skolnick [8] developed a clustering approach, called SCAR, that uses a harmonic potential to refine centroid structures. However, structure prediction results indicate that SPICKER [9] outperforms SCAR in terms of model selection. Furthermore, it has been shown [10] that 'the models generated by TASSER [11] have incorrect side-chain conformations and poor hydrogen bonding patterns partly because of the on-lattice modelling and the unphysical geometry of the SPICKER[9] cluster centroid structure'. PULCHRA [1], which combines a conjugant gradient search with a harmonic potential, supersedes both SCAR and SPICKER. Similarly, Kolinski and Bujnicki [12] have introduced an elegant approach using a combination of template-based and *de novo *modelling followed by hierarchical clustering that employed averaging of very diverse models from threading, that results in consensus structures with improved local and global quality.

In general, averaging artifacts become more pronounced when members of the ensemble are more divergent. This artifact is exacerbated in TASSER due to the fact that it begins with a lattice model and averaging is performed across clusters of dissimilar structures. Consequently, the averaged structure is often not suitable for detailed atomic model building due to unrealistic bond lengths and angles and unphysical local geometry. In the same vein, the community wide experiment on the Critical Assessment of Techniques for Protein Structure Prediction (CASP) also penalizes structures with unphysical bond lengths and unrealistic geometries. CASP defines two types of clashes for bond length. The first type of clash involves atoms that are less than 1.9 Å apart, and the other type of clash involves atoms that are less than 3.6 Å apart. We adopt these criteria in what follows.

Herein, we apply the proposed algorithm for removing averaging artifacts from clusters of structures generated by the TASSER (Threading/ASSembly/Refinement) algorithm [11]. Within TASSER, generated structures are clustered using SPICKER [9] and the cluster with the highest structural density is selected (this is still true in current version of TASSER [10]). Subsequently, the centroid model (called COMBO) that is obtained by averaging all the cluster members of the most densely populated cluster is selected as the predicted structure. In various benchmarks of the TASSER algorithm [13], the averaged structure (aka, the COMBO model) is generally closest to the native in terms of global RMSD. It is closer to the native structure than all the individual cluster members, including the medoid (CLOSC model). Hence, TASSER outputs the cluster centroid (COMBO model) as its final model. In this regard, averaged models have also been shown to outperform minimum free energy structures in the context of RNA secondary structure prediction [2].

Our goal is to generate a structure that is as close as possible to the 'averaged structure' while maintaining realistic bond lengths and angles and local geometry. Unless otherwise stated, the term bond length refers to 'virtual bond length' between two Cα atoms throughout this report, and bond angle refers to 'virtual bond angle' between any three consecutive Cα atoms. To address this issue, we have developed a new algorithm, MCORE (**M**onte **C**arl**O **based **RE**finement) that is designed to generate such structures by minimizing the difference between the 'averaged' and the physically reasonable structure using a Monte Carlo minimization procedure. We show that our approach is robust and general and can overcome averaging artifacts with minimal reduction of structure quality as assessed by the RMSD of the resulting model from the native structure. Once the refined Cα model is obtained, then approaches like the one based on Backbone Building from Quadrilaterals proposed by Gront et al. [14] can be used to complete backbone reconstruction.

## Methods

The central idea behind our approach is to start from a structure that has physically allowed bond lengths and then minimize the difference between this starting structure and the averaged structure. In this respect, our methodology consists of two basic components: (1) generation of the starting structure and (2) minimization of this starting structure in the presence of the averaged structure.

### Starting Structure

We explore two types of starting structures: (1) a fully extended structure with bond length corresponding to the average bond length obtained from the PDB and all ψ and ψ = 180°, (2) a model that is close to the 'averaged structure' but has physically reasonable bond length and angles, which we call the 'close-by model'. A typical model of this type can be the structure that is closest (based on RMSD) to the 'averaged structure' in an ensemble of proteins. In case of TASSER, CLOSC models fall in this category. In case, when two structures have the same RMSD to the averaged structure, one of them is chosen at random. Extended structures will be required when no 'close-by model' is available.

### Energy Function

The pseudo energy potential, *V*, in our algorithm is presented in equation (1). The potential, *V*, consists of three components: a harmonic term for excluded volume violations, a harmonic term for virtual bond angle violations and a third term that drives the conformation towards the target structure. Thus, *V *is given by

(1) $$V=k_{excl}\sum_{k=1}^{N-2}\sum_{l=k+2}^{N}{\left(r_{kl}-r_{0\_excl}\right)}^{2}+k_{ang}\sum_{k=1}^{N-2}{\left(\theta_{i,i+1,i+2}-\theta_{0\_ang}\right)}^{2}+k_{clos}\sum_{k=1}^{N}{\left(d_{kk_{t}}-d_{0\_clo}\right)}^{2}$$

where *N *is the number of Cα atoms; *k*_*excl*_, *k*_*ang*_, *k*_*clos *_are the weights of corresponding contributions to *V*. *r*_*kl *_is the distance between the *k*^*th *^and *l*^*th *^Cα atoms, *r*_0_*excl *_is the cutoff parameter for excluded volume violations and is set to 4 Å if *r*_*kl *_< 4.0 Å, otherwise *r*_0_*excl *_is set to be equal to *r*_*kl*_. That is, this contribution to the potential is turned off. θ_*i*, *i*+1, *i*+2 _is the virtual bond angle formed by the *i*^*th*^, *i+1*^*st*^, and *i+2*^*nd *^Cα atom θ_0_*ang *_is the cut-off angle, and is set to be 70° if θ_*i*, *i*+1, *i*+2 _< 70°, 150° if θ_*i*, *i*+1, *i*+2 _> 150°, or θ_0_*ang *_= θ_*i*, *i*+1, *i*+2 _otherwise; $d_{kk_{t}}$ is the distance between the corresponding Cα atoms of the target structure and the current conformation, and *d*_0_*clo *_is the maximum allowed displacement between the corresponding Cα atoms and is set to be equal to 0.001 if $d_{kk_{t}}$ > 0.001 Å or is set equal to $d_{kk_{t}}$ otherwise. The values for these parameters are chosen such that they are close to those by Oldfield et al. [15] The values of *k*_*clos*_, *k*_*excl*_, and *k*_*ang*_are chosen to be 1.0831, 0.56818 and 0.015, respectively, on the basis of optimization of the parameters using MINUIT [16] to maximize the correlation between the energy function and the RMSD to the native structure and manual adjustment based on empirical observation for a set of 726 proteins that are used for training parameters as described in the data set. The RMSD values are measured on the Cα atoms for all the cases except those where specified.

### Move Sets

**Figure 1:**
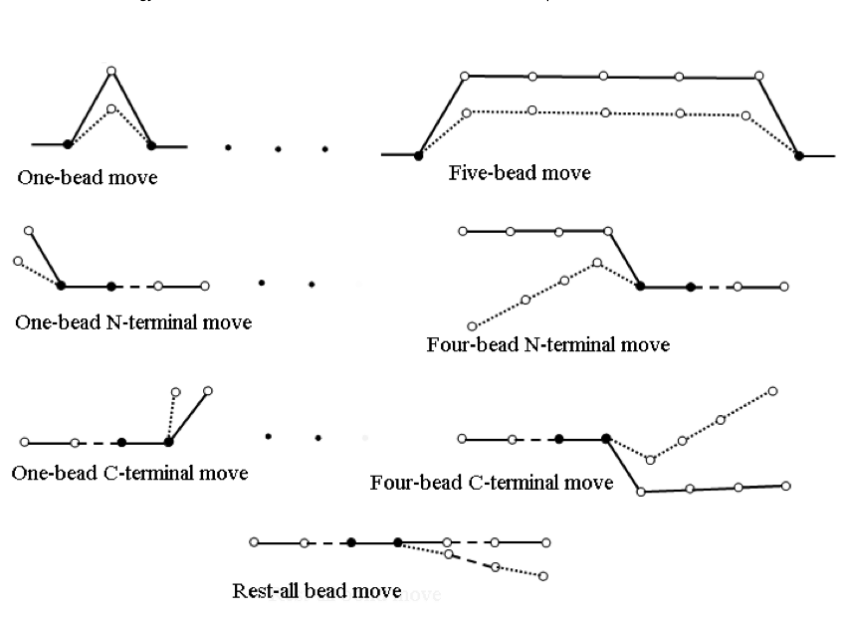
**Schematic diagram of move sets**. Illustration of different move sets. The circles represent Cα atoms. The axis joining two black circles in each figure represents the axis of rotation of all other involved atoms. The solid line represents the orientation of the Cα atoms before the move and dotted lines represent the orientation of the Cα atoms after the move. The middle dots represent the same figures for two-bead, three-bead and four-bead moves in case of the top row, two-bead and three-bead moves in case of 2^nd ^and 3^rd ^row figures respectively. The move sets in the first three rows are 'local moves' and the 'Rest-all bead move' is a global move.

Another important aspect of a Monte Carlo simulation is the *move set *that moves the structure from the current conformation to the next one. Selection of move sets is very critical to the performance of the simulation itself. We have designed two types of move sets, one of which is global and another is local. Both sets preserve initial bond lengths. A schematic overview of both is depicted in Figure 1. There are two types of local moves: i) one to five bead moves that preserve the geometry of the chain outside the fragment whose conformation is changed and ii) one to four bead moves at both ends of the chain. In both the cases, the geometry of the chain outside the targeted fragment is preserved. The global move involves a global rotation of the entire chain, which for the *i*^*th *^residue involves a rotation about the *i*-1 to *i*^*th *^bond. A given Monte Carlo step consists of *N-k*-1 attempts at a *k*-bead move (where *k *= 1 to 5), plus *k *(= 5) attempts at each of *l*-bead N-terminal and *l*-bead C-terminal moves (where *l *= 1 to 4), and one attempt at a global reorientation move. Of course, attempt locations are randomly chosen.

**Figure 2:**
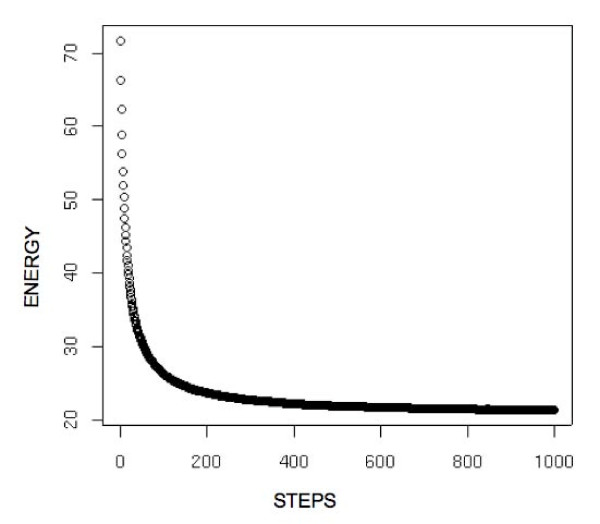
**Snapshot of energy Vs Number of steps**. The snapshot of energy Vs Number of Monte Carlo steps for driving the corresponding extended structure to its native structure for a set of 726 proteins as defined in the data set.

We performed computational experiments on the set of 726 proteins described below where an extended structure (which has identical bond lengths to the native from which it was generated) was driven towards the corresponding average structure using the algorithm described above. The snapshot of energy vs. number of steps for this set of experiments is shown in Figure 2. The average Cα RMSD of the proteins to their respective native structure for a relatively short (1000 steps) run was 0.06 Å.

### Convergency Criteria

There are no straightforward convergence criteria for Monte Carlo Simulations (MCS). However, two obvious convergence criteria are: (1) allowing for a pre-specified total number of steps and (2) allowing the algorithm to proceed until it ceases to make progress. Herein, we use both types of convergency criteria. Starting from the 726 extended structures, the average final RMSD for a 2000 steps run was 0.05 Å. Hence, we choose 2000 steps as the specified steps for our simulation. Furthermore, we also devised a mechanism to stop the algorithm when it ceases to make progress. We define that the algorithm ceases to make progress after step *j *if following criteria is satisfied for every *i*, where *1 *<*i *<*n*:

(2) ∀_*i*_(*RMSD*_*j *_- *RMSD*_*j*-*i*_) <*T*

where *RMSD*_*j *_is the RMSD of the conformation after *j *steps and *RMDS*_*j*-*i *_is the RMSD of the conformation after *j-i *steps. The value of *i *goes from 1 through *n *and *T *is the tolerance cutoff. Once the step *j *(where the algorithm ceases to make progress) is obtained, the simulation is run for extra *x *steps. In other words, the simulation is stopped after *j*+*x *number of steps if the value of RMSD of the last *n *steps is within the tolerance region compared to the value of the step *j*. The values of *n*, *x *and *T *were chosen empirically and were chosen to be 50, 10 and 0.05 respectively. Monte Carlo simulations were performed using the above move set and the standard Metropolis criteria at a temperature of 450 K [17].

### Data Set

To verify the application of MCORE algorithm, we use it to remove the averaging artifacts in the output of TASSER algorithm. The data set used for this study consists of 2090 non-homologous single domain proteins with less than 200 residues with a maximum of 35% pairwise sequence identity to each other that cover the Protein Data Bank. All of these proteins have an initial RMSD of COMBO model (averaged model) against the native protein to be less than 6.5 Å. This is from the fact that the predicted models that are about 6.0 Å to the native structures are likely to have the same fold as the native structure [18]. In addition, from the TASSER outputs we have corresponding COMBO structure and CLOSC structures for each of these proteins. Out of the 2090 proteins, 726 proteins are used for training of model parameters, whereas the remaining 1364 proteins are used for validation. All root mean square deviation (RMSD) values refer to Cα atom comparisons unless otherwise stated.

## Results and discussion

### Comparison of Two Types of Starting Structures

For the comparison between the two types of starting structure schemes: extended structure and 'close-by' model, we performed computational experiments on the set of 1364 test set of proteins as described in the data set. For each type of starting structure scheme, we run our algorithm for 100, 200 and 2000 steps (the results are presented in Table 1). It can be observed from the table that starting from close-by models produce better results in all three regimes (i.e., 100, 200 and 2000 steps) relative to the extended models. Hence, for the comparison of our method with CLOSC and COMBO models, we use the close-by starting scheme. The algorithm with close-by starting scheme that uses convergency criteria as described in equation 2 is termed MCORE, whereas the version of the algorithm with fixed number of steps (= 2000) using the same close-by starting scheme is termed MCORE-L.

**Table 1:** Comparison of results for two types of starting structures^1^

*Starting Structure*	*100 steps*	*200 steps*	*2000 steps*
*Extended*	1 min/3.38 Å/1.8%	2 min/3.35 Å/1.5%	20 min/3.34 Å/1.3%
*'Close-by model'*	1 min/3.36 Å/1.0%	2 min/3.35 Å/1.0%	20 min/3.34 Å/1.2%

^1^Extended and 'close-by model' for 100 steps, 200 steps and 2000 steps for the same set of 1364 proteins. The numbers X/Y/Z denote time required (on a Desktop pc with 2 GB of RAM and 2.0 G Hz Intel processor) for the respective runs, RMSD to the native and the percentage of atoms that are involved in clashes less than 3.6 Å respectively.

### Refinement of COMBO Models

Before comparing the results of the refined models, we also take an opportunity to analyze the RMSD to native of the 1364 COMBO and CLOSC models. Across the dataset, only 100 CLOSC models had lower RMSD values relative to the native structure compared to the COMBO models, reiterating the advantage of averaged structures in this regard. The average RMSD of COMBO model to the native structure is 3.28 Å, whereas the average RMSD of CLOSC model to the corresponding native structure is 3.55 Å.

**Figure 3:**
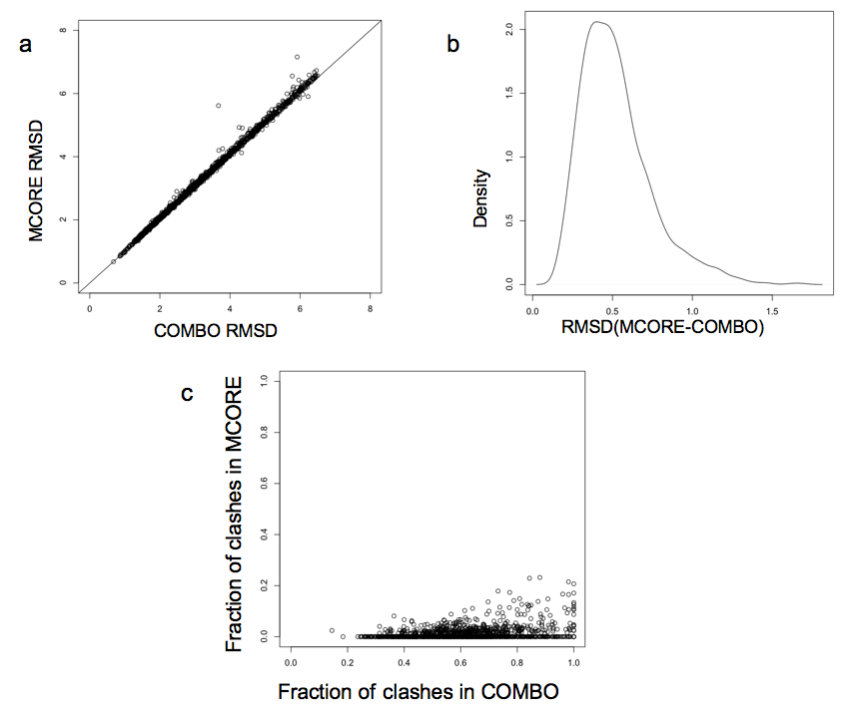
**Comparison of COMBO models and MCORE models**. (a) Scatter plot of the Cα RMSD of combo models and respective MCORE refined models for a set of 1364 proteins compared to corresponding native structure (b) Density plot of distribution of RMSD deviations between MCORE structures and the corresponding COMBO structures. c) Scatter plot of the fraction of atoms involved clashes in COMBO models and corresponding refined models for the same set.

Upon application of the MCORE algorithm to the refine the COMBO models, it is found that the refined representative structures have similar RMSD values to native structures, but with far fewer unphysical characteristics. Figure 3(a) plots the RMSD values of the MCORE to native comparisons versus the COMBO to native comparisons, which demonstrates a strong linear correlation between the two methods. The RMSD values of the COMBO models are only slightly better than those from MCORE. This point is reinforced by Figure 3(b), which plots the density of RMSD differences between the methods. The majority of RMSD differences are slightly less than 0.5 Å. In addition, 38 MCORE refined structures had even better RMSD than their corresponding COMBO model. Figure 3(c) plots fraction of clashes within the MCORE vs. COMBO models. Clearly, the MCORE models have far fewer clashes than their COMBO counterparts. The average percentage of clashes in MCORE refined models is 1.09%, whereas the average percentage of clashes in COMBO models is 63.0%.

We also compared the RMSD (to native) of the MCORE refined COMBO models to that of the unrefined CLOSC models. Here, it was observed that only 99 of 1364 MCORE refined models had poorer RMSD values than the corresponding CLOSC models. The average RMSD for MCORE models was 3.36 Å. In addition, for MCORE-L, we were able to obtain an average RMSD of 3.35 Å. Based on the much reduced compute time of the MCORE algorithm (discussed below), it is satisfying to note that the average RMSD of MCORE-L models and MCORE models are virtually the same. These results are summarized in Table 2.

**Table 2:** Cα RMSD of MCORE and other Models compared to the native structure for a set of 1364 proteins in terms of RMSD, TM-SCORE and percentage of atoms in the clashes^2^.

	*Methods*	*Average*	*Std. Dev*
*RMSD*(Å)	COMBO	3.28	1.39
	PULCHRA	3.35	1.77
	CLOSC	3.55	1.49
	MCORE	3.36	1.42
	MCORE-L	3.35	1.41
	*Methods*	*Average*	*Std. Dev*
*TM-Score*	COMBO	0.743	0.13
	PULCHRA	0.746	0.13
	CLOSC	0.719	0.13
	MCORE	0.744	0.13
	MCORE-L	0.746	0.13
	*Methods*	*Average*	*Std. Dev*
*Clashes *<*1.9*	COMBO	4.50	10.01
	PULCHRA	0.05	0.4
	CLOSC	0	0
	MCORE	0	0
	MCORE-L	0	0
	*Methods*	*Average*	*Std. Dev*
*Clashes *<*3.6*	COMBO	63.40	18.67
	PULCHRA	3.64	12.00
	CLOSC	0.00	0
	MCORE	1.09	3.69
	MCORE-L	1.20	4.02

^2 ^The corresponding standard deviation values are also provided.

Overall, it is found that MCORE produces models better in terms of RMSD and TM-score [19] versus the corresponding CLOSC models. Moreover, the MCORE models are only slightly worse than the averaged COMBO models, which is consistent with our initial problem statement. Note that the larger TM-score, the better the model. Moreover, if we discriminate clashes into the two CASP types, then it is more evident that our refined models are much better in terms of clashes as they do not have any atoms involved in clashes that are less than 1.9 Å, whereas COMBO models have 4.5% of the atoms involved in this regime of clashes.

We also investigated the average number of steps and time for the MCORE and MCORE-L algorithms. It was observed that for MCORE the number of average Monte Carlo Steps is less than 110 (= 109.21) and the average running time is 1.88 minutes. Moreover, for MCORE-L, the average running time is 20 minutes. Hence, MCORE can be applied to a wide variety of problems concerning the averaging of macromolecular structures due to its fast execution time.

**Figure 4:**
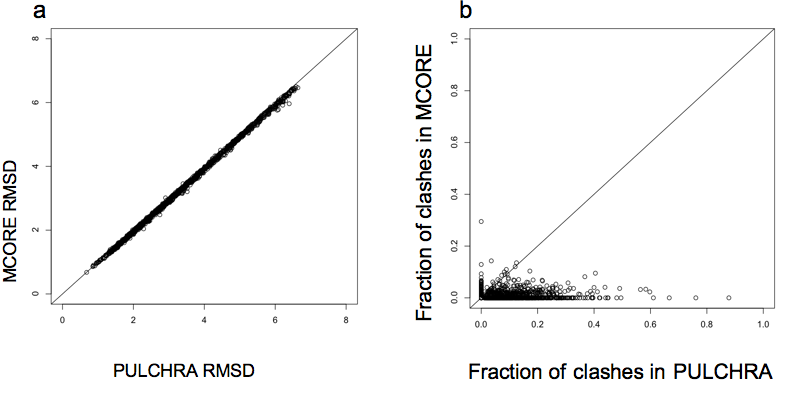
**Comparison of PULCHRA models and MCORE models**. (a) Scatter plot of the RMSD of PULCHRA models and respective MCORE refined models for a set of 1364 proteins (b) Scatter plot of the total number of clashes in PULCHRA models and corresponding refined models for the same set.

We also analyzed some representative proteins that have higher RMSD deviation compared to the COMBO structure. In Figure 4(a) we present the COMBO and native model and in Figure 4(b) we present the MCORE and native model of protein 1QLE (chain D) which had the largest RMSD deviation compared to the COMBO structure. Furthermore, to highlight the differences, we magnify the N-terminus region of Figures 4(a) in 4(c) and 4(b) in 4(d) respectively. As can be seen in the figure, the N-terminus region of the cytochrome C oxidase of the protein in the COMBO model is totally unphysical and hence, the large RMSD deviation between the MCORE models and COMBO models. We also analyzed the virtual bond distance in the model and found that there were two bonds less than 0.8 Å and 9 bonds less than 2.5 Å. We also analyzed other representative structures. As suspected, we found that in most of the cases where there was a large deviation between the COMBO RMSD and MCORE RMSD, there was involvement of unphysical bond lengths which reiterates the fact that there is trade-off between local geometric correctness and deviation from the target structure.

### Comparison with PULCHRA

**Figure 5:**
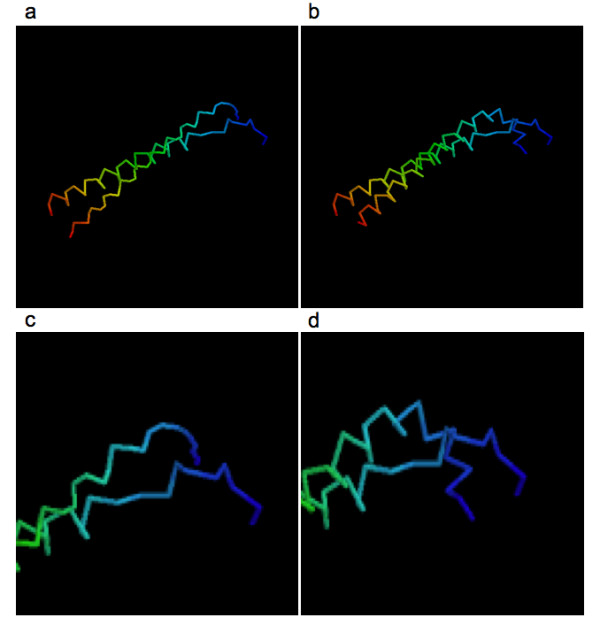
**Representative Rasmol view of a PDB (1QLE:D) where the RMSD of the MCORE model and the corresponding COMBO model was the highest**. a) The COMBO model compared to the native structure. b) Refined model compared to the native structure, c) magnified N-terminal region of Figure 4(a) and d) Magnified N-terminal region of Figure 4(b). The coloring is based on RasMol default coloring where N terminus is colored red and the C terminus are colored blue.

For the comparison of MCORE algorithm with existing approaches, we also compared our results with PULCHRA [1] refinement. PULCHRA is an all atom reconstruction method that has an optimization of Cα carbon position using steepest descent minimization procedure. Figure 5(a) plots the RMSD values of the PULCHRA to native comparisons versus MCORE to native comparison. Figure 5(b) plots the fraction of atoms involved in clashes less than 3.6 Å in PULCHRA models versus the MCORE models. The average RMSD of MCORE refined COMBO models was found to be 3.36 Å as compared to 3.35 Å for PULCHRA. However, in terms of clashes MCORE models on average only have 1.09%, whereas 3.64% of the atoms from the PULCHRA models are involved in clashes. This difference in clashes is statistically significant as shown in Table 3 using a standard Z-test. Moreover, if we break down the clashes into clashes less than 1.9 Å and clashes less than 3.6 Å, it is found that MCORE models do not have any clashes less than 1.9 Å, whereas PULCHRA does (see Table 2). The clashes less than 1.9 Å are severe for further refinement of the models. While the MCORE models are slightly worse than those from PULCHRA in terms of RMSD to native by 0.01 Å, they have a statistically significant improvement in terms of clashes. Moreover, 480 (out of the 1464) of the MCORE models resulted in better refinement of the COMBO model versus PULCHRA. In addition, for long runs of MCORE (MCORE-L), we were able to obtain an average RMSD of 3.35 Å, which is exactly same as obtained by PULCHRA. Moreover, the MCORE-L models had far fewer clashes than the PULCHRA models (1.2% vs. 3.64%, respectively).

**Table 3:** Comparison of MCORE to COMBO and to PULCHRA in terms of RMSD and percentage of atoms involved in the clashes^3^.

*Combo Vs MCORE*		
*RMSD *(Å)	Combo	3.28
	MCORE	3.36
	p-value	0.008
*Clashes*	Combo	63.00%
	MCORE	1.09%
	p-value	**< 2.22E-16**
*MCORE Vs PULCHRA*		
*RMSD *(Å)	PULCHRA	3.35
	MCORE	3.36
	p-value	0.017
*Clashes*	PULCHRA	3.64%
	MCORE	1.00%
	p-value	**2.22E-16**

^3^P-values are given where available using the z-test. Significant P-values are in bold.

Furthermore, MCORE algorithm is comparable to PULCHRA in terms of efficiency also, as on average the computation time is around a minute. One of the major advantages of our approach compared to PULCHRA is that if the input structure is heavily distorted, PULCHRA might fail to converge where MCORE will always converge.

### All-atom Model Reconstruction

It is essential to have a model with physical bond lengths and bond angles if further analysis is to be performed on the model. Since structure prediction methods often produce Cα-only models, all-atom models must be constructed from the Cα descriptions. In this regard, we built all-atom representations of the MCORE refined Cα atom models. The initial backbone reconstruction method applied is the backbone reconstruction method of an algorithm proposed by Milik et al [20]. Once the backbone atoms are reconstructed, any side-chain packing methods [21,22] can be utilized to build the side-chains. We performed the side-chain reconstruction using one of the most widely used side-chain packing algorithms SCWRL 3.0 [22]. The MCORE refined models for the set of 1364 proteins had an average all-atom RMSD of 4.19 Å (which is, or course, higher than the value of 3.35 Å for the Cα models). The PULCHRA refined all-atom models on the same dataset had a comparable average value of 4.17 Å.

## Conclusion

In this paper, we presented MCORE, a Monte-Carlo based algorithm for removing averaging artifacts of the averaged structure to improve the quality of the consensus structure. We verified the application of the proposed algorithm by applying the algorithm to refine the COMBO models of a set of 1364 proteins generated by TASSER algorithm, refining and correcting unphysical bond length and bond angles. On average, the RMSD to native of the refined model is 3.36 Å; where as RMSD of the COMBO model to the native is 3.28 Å, which is a mere 0.08 Å poorer than the RMSD of the COMBO model (averaged model). On the other hand, the average percentage of atoms involved in the clashes in the refined MCORE models is reduced from 63% (for the COMBO models) to only 1.0%. Moreover, slight RMSD gains were obtained by using a version of the MCORE algorithm that samples longer. However, the difference between MCORE-L (the longer version) and MCORE (Table 3) is not statistically significant, emphasizing that our convergence criterion is robust.

We have also generated a framework for producing all-atom models from the Cα only atom models by first reconstructing the backbone and then doing the side-chain reconstruction using existing methodologies. An obvious extension of the work is to refine not only Cα models, but also to apply MCORE to all-atom models. In essence, the new refinement algorithm helps in attaining structures with more physical bond lengths and bond angles by overcoming averaging artifacts produced due to averaging of structures. It has to be noted that there is always a trade-off between local geometric correctness and the deviation from the target structure. Generating averaged structures that are not heavily distorted can minimize this trade-off. These results provide a genuine model for the subsequent analysis of the respective protein structure using molecular mechanics force field. In addition, this algorithm does not have convergence problems like PULCHRA (which sometimes fails to converge if the input models are heavily distorted). Although the algorithm was tested for TASSER models only, the presented approach is general and can be applied to remove averaging artifacts arising from averaging over any ensemble of molecular conformations.

## Abbreviations

RMSD: Root Mean Square Deviation; TASSER: Threading/ASSembly/refinement; CASP: Critical Assessment of Techniques for Protein Structure Prediction; NMR: Nuclear Magnetic Resonance; MCORE : MOnte Carlo based Refinement; COMBO model: Centroid model; CLOSC model: Model which is closest to the centroid Model.

## Authors' contributions

DBKC wrote the program and carried out the experiments and authored the manuscript. All authors read and approved the final manuscript.
